# Efficacy of Four-Channel Functional Electrical Stimulation on Moderate Arm Paresis in Subacute Stroke Patients—Results from a Randomized Controlled Trial

**DOI:** 10.3390/healthcare10040704

**Published:** 2022-04-10

**Authors:** Thomas Schick, Daniela Kolm, Andreas Leitner, Sandra Schober, Maria Steinmetz, Klemens Fheodoroff

**Affiliations:** 1 MED-EL, Medical Electronics, Business Unit Neurorehabilitation STIWELL, Fürstenweg 77a, 6020 Innsbruck, Austria; 2 KABEG Gailtal-Klinik, Neurorehabilitation, Radnigerstrasse 12, 9620 Hermagor, Austria; daniela.kolm@kabeg.at (D.K.); andreas.leitner@kabeg.at (A.L.); sandra.schober@kabeg.at (S.S.); klemens.fheodoroff@kabeg.at (K.F.); 3 Therapy Center Riedhart, Occupational Therapy, Innsbrucker Str. 9, 6300 Wörgl, Austria; steinmetz.maria@gmx.at

**Keywords:** moderate arm paresis, subacute stroke, motor recovery, control of voluntary movements, EMG-triggered multichannel electrical stimulation, functional electrical stimulation

## Abstract

This preliminary randomized clinical trial explores the efficacy of task-oriented electromyography (EMG)-triggered multichannel functional electrical stimulation (EMG-MES) compared to single-channel cyclic neuromuscular electrical stimulation (cNMES) on regaining control of voluntary movements (CVM) and the ability to execute arm-hand-activities in subacute stroke patients with moderate arm paresis. Twelve ischemic stroke patients (Fugl-Meyer Assessment Arm Section (FMA-AS) score: 19–47) with comparable demographics were block-randomized to receive 15 sessions of cNMES or EMG-MES over three weeks additionally to a conventional neurorehabilitation program including task-oriented arm training. FMA-AS, Box-and-Block Test (BBT), and Stroke-Impact-Scale (SIS) were recorded at baseline and follow-up. All participants demonstrated significant improvement in FMA-AS and BBT. Participants treated with EMG-MES had a higher mean gain in FMA-AS than those treated with cNMES. In the SIS daily activities domain, both groups improved non-significantly; participants in the EMG-MES group had higher improvement in arm-hand use and stroke recovery. EMG-MES treatment demonstrated a higher gain of CVM and self-reported daily activities, arm-hand use, and stroke recovery compared to cNMES treatment of the wrist only. The protocol of this proof-of-concept study seems robust enough to be used in a larger trial to confirm these preliminary findings.

## 1. Introduction

Stroke is the main reason for disability in adults [1]. Neurorehabilitation focuses on regaining control of voluntary movements (CVM) to optimize task performance in activities of daily living, thus improving quality of life. Houwink et al. [2] found that 78% of stroke patients with an initial CVM in the shoulder and elbow at admission to first in-patient rehabilitation achieved advanced arm and hand capacity after discharge.

Recovery of CVM in paretic arm and hand (with or without synkinesis) can best be quantified with the Fugl-Meyer Assessment Arm Section (FMA-AS; score 0–66) [3,4]. 

In recent years, electrical stimulation (ES) of an upper extremity is demonstrated to be a promising intervention to improve arm and hand function in neurorehabilitation [5,6]. Data have been published on the effects of both cyclical and electromyography (EMG)-triggered single-channel ES [6,7,8]. The research exhibits the positive effects of Functional Electrical Stimulation (FES) to improve the ability to perform activities [6,8].

Interventions that support voluntary movements through an ES triggered by an EMG signal, which is induced by a target movement performed from the affected individual, are increasingly gaining acceptance. This type of intervention was described as even more reinforcing than cyclic stimulation due to the proprioceptive feedback and voluntary component involved. Motor improvements in hand function following a stroke have been observed [9,10,11,12]. A meta-analysis describes the comparison of EMG-triggered ES to conventional therapy and demonstrates improved upper limb impairment in those more than 3-month post stroke. The benefits within the first month and the comparison of the effects of different EMG-stimulation protocols are not clear yet. These authors suggested that EMG-ES leads to greater gains in post stroke upper extremity impairment [7]. In a study on stroke patients, EMG-ES was compared with classical transcutaneous nerve stimulation (TENS) below the motor threshold. EMG-triggered stimulation leads to better short-term effects in the intervention group [13]. In contrast to a pure repetition of movement in one joint, functional multi-joint arm-and-hand activities can be elicited by EMG-triggered multichannel stimulation patterns (EMG-MES) [11,14,15]. Patients with moderate arm paresis can use their residual CVM to trigger voluntary movements [7,16]. A higher activity level of the patient has been proposed as an additional advantage for this type of intervention. 

EMG-MES has also been studied as part of motor re-learning programs and for arm-and-hand use in stroke patients [11,12,14,15]. As functional electrical stimulation (FES) is defined as a process of pairing stimulation simultaneously or intermittently with a functional task [17], EMG-MES is a variation of FES through its functional and target oriented approach [12].

Although the effects of ES suggest improvement in arm function in stroke patients, it is not yet clear which procedure and parameter choice of different forms of electrical stimulation (sensory afferent stimulation; cyclic electrical stimulation; EMG-triggered electrical stimulation) is superior [6,8].

Currently, it is unclear which type of stimulation (and which movements) should be used for different severity levels of impaired CVM. There are few data indicating the superiority of EMG-MES compared with cNMES [16,18]. The study presented in this article should provide additional insight on this topic.

## 2. Materials and Methods

### 2.1. Study Design and Participants

This was a randomized, controlled, single-assessor blinded study conducted in a stroke in-patient neurorehabilitation center in Austria.

The study was approved by the Ethics Committee of the State of Carinthia, Austria (vote A06/18—26 April 2018), and registered at the German Registry for clinical trials (DRKS) under the national clinical trial registration ID number DRKS00014358 and conforms to the ICMJE guidelines. 

Relevant inclusion and exclusion criteria were:

### 2.2. Inclusion Criteria

- First ischemic stroke with moderate arm paresis * (19 ≤ FMA-AS ≤ 47) [19];
- Existing ADL capability before the event;
- Early and late post-acute phase 1–6 months [20];
- Age between 18 and 80 years;
- Capacity for written informed consent.

### 2.3. Exclusion Criteria

- Implanted defibrillators, brain stimulators, pacemakers, and drug pumps;
- Severe contractures in the treatment area;
- Wounds in the stimulation area;
- Pregnancy.

Participants’ independent execution of movements in activities of daily living (ADL) and full arm-hand activities prior to the stroke were determined by asking participants to describe their history of ADL and arm-hand activities before entering the study. Written informed consent was acquired from all participants or their legal custodians’ previous study entry. Strict data security was ensured during and after the study. The entire study documentation was anonymized and transferred to the study investigator for safekeeping. Personal data remained at the responsible rehabilitation center and will continue to be saved on-site during regular archiving. Video recordings were stored on a password-protected online database and made available to the external assessor. Videos were deleted from that database after evaluation and saved to a secure external hard drive by the investigator. Subjects were informed about the two test groups but not about the study hypothesis.

### 2.4. Randomization

Participants were block-randomized to the intervention group (IG) or the control group (CG) in blocks of six participants for each group. In each block, three participants were allocated to the IG and the CG. Before study initiation, the study manager provided two master envelopes to the person responsible for study execution. Each master envelope contained six numbered and sealed envelopes. Each of the sealed envelopes included one of the random numbers generated by Microsoft Excel^®^ (Version 2016) (Santa Rosa, CA, USA), the resultant randomized allocation to the IG or CG, and all tests, procedures, and relevant documents that were needed to carry out the study. The centers were not allowed to open the second master envelope until all envelopes of the first master envelope were used (block randomization) [21,22]. All envelopes were numbered and drawn by a blinded person who was not directly involved in the study. With this process, a number and a group were assigned to each participant. 

### 2.5. Intervention

All study participants underwent intensive arm-hand training as part of their in-patient stroke rehabilitation program (training of repetitive (single and complex) movements and task-oriented training within given capacity and muscle endurance, 5 × 30 min per week). During the three-week study period, the participants received 15 training sessions with either EMG-MES or with cNMES (5 × 30 min per week) in addition to their conventional rehabilitation program. The study duration was 3 weeks with 5 interventions per week (30 min each) of the respective additional treatment options, with participants seated on a chair in front of a height-adjustable desk that was adapted to their individual heights. 

ES in both groups was performed with the STIWELL^®^ med4 device (CE 0297; P/N 9001015) developed by MED−EL Elektromedizinische Geräte Gesellschaft m.b.H., Innsbruck, Austria). This electrical stimulation device has four muscle stimulation channels and up to two channels for EMG signal uptake. For all study participants, the same type of surface electrodes (PALS^®^ Neurostimulation electrodes, oval 4 cm × 6.4 cm, Axelgaard Manufacturing Co., Ltd., Lystrup, Denmark, CE-certified, REF 896230) were used. 

In both groups, rectangular biphasic pulses with a pulse width of 300 µs were used. A pulse intensity (mA) to elicit a sufficient and possibly visible grip motion of the affected limb was selected according to the participants’ level of tolerance. The standard current frequency was 30 Hz.

Since the differences in the procedures of the two therapy options were quite apparent (EMG-MES versus cNMES), therapy sessions that were blinded for both the therapists and the participants were not possible. A single-blinded study design with blinded video assessments (as described below) was chosen for this reason. 

### 2.6. EMG-MES

The therapy sessions of the IG consisted of task-oriented unilateral arm-and-hand movements on the affected side with four-channel EMG-triggered pulses for reaching, gripping, and lateral lifting of cylindrical objects to different heights (0 cm, 6 cm, 12 cm, and 18 cm), dependent on each participant’s improvement in dexterity and range of motion. To optimize participants’ movement response, the correct electrode position was inspected and corrected by experienced therapists before each intervention. During any session and throughout the whole treatment period, therapists were allowed to initially support the movement slightly if necessary.

**Figure 1:**
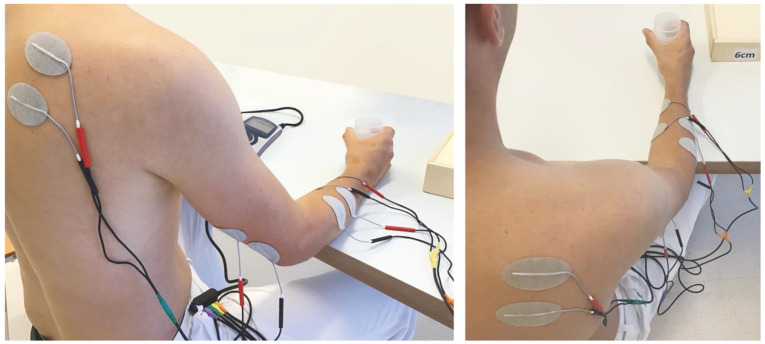
Model demonstrating electrode placement for the 4-channel EMG-triggered pulses for reaching, gripping, and lateral lifting of cylindrical objects to different heights.

Electrode pairs in the IG were placed on the participant’s arm and/or hand according to the required task and the superficial muscle groups involved in this movement. Channel 1 was used for wrist extension, channel 2 for finger flexion, channel 3 for external rotation in the shoulder joint, and channel 4 for elbow extension (preferably M. extensor carpi radialis longus or brevis, M. flexor digitorum superficialis, M. infraspinatus, and M. triceps brachii). See Figure 1.

**Figure 2:**
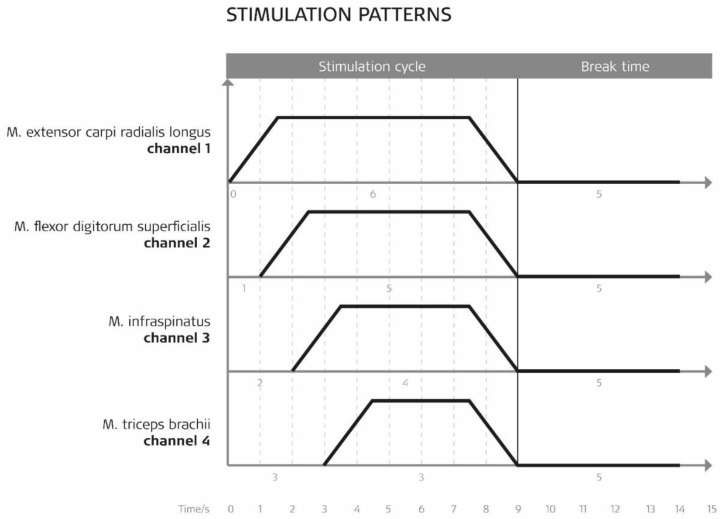
Stimulation pattern for EMG-MES with contraction and pause time, rise and fall time, and the stimulated muscles on the respective channels.

The standard value for this complex four-channel activity for the first channel was a 6-s stimulation plateau time and a 5-s pause time. With a delay of 1 s, the second channel followed with a plateau of 5 s. Channel 3 had a delay of 2 s (compared to channel 1) and a 4-s plateau time followed by channel 4 with a 3-s delay time (compared to channel 1) and a 3-s plateau time. See Figure 2.

The increase and decrease times were 1.5 s, respectively. If necessary, the standard values were adapted to individual needs. 

The multichannel electrical stimulation (MES) was triggered by surface electrodes that detected the initial EMG signals for the affected target muscle group (default: wrist extensors). After reaching an individually defined threshold, the electrical stimulation pattern was delivered as described in the previous section. The threshold was defined in such a way that the patient could reach it without involuntary co-contractions while performing the task during his movement initiation. This ensured, among other things, that the subject was not only active during reaching the threshold of the EMG-triggered impulse release but also during the active movement implementation of the required task.

### 2.7. cNMES

In the CG, the pair of electrodes was placed on the wrist extensor muscle (M. extensor carpi radialis longus) of the affected side. According to a passive cyclic stimulation, no EMG trigger was used. A clear dorsal extension movement in the wrist should be visible during stimulation. The standard value for this activity had an on/off time cycle (on 6 s/off 14 s) but was adjusted for each individual participant if needed. This test setup did not allow any additional active task-oriented therapy under the stimulation.

### 2.8. Outcome Measures

The following assessments were performed before and after the treatment:

- Fugl-Meyer Assessment Arm Section Score (FMA-AS)—blinded video rating;
- Box and Block Test (BBT);
- Stroke Impact Scale (SIS)—German version.

As the primary outcome of the study, each participant’s total FMA-AS score (0–66 points including reflexes and pain) was calculated and analyzed [23]. Higher scores indicate better function. Four FMA-AS subscales were also analyzed (A: upper extremity, 0–36 points; B: wrist, 0–10 points; C: hand, 0–14 points; and D: coordination/speed, 0–6 points). The FMA-AS was videotaped following a standardized protocol and the footage was assessed by an external blinded assessor (M.S.)

The BBT score [24] of each participant was collected by the therapists to assess participants’ manual dexterity in object manipulation. Higher scores indicate better manual dexterity.

As a patient-reported outcome measure, the German version of the SIS was recorded within the week before and within the week after the intervention. This instrument contains 64 items on a Likert scale and assesses eight dimensions of health-related quality of life (“strength”, “memory and thinking”, “emotion”, “communication”, “ADL/IADL”, “mobility”, “hand function”, “participation”, and “stroke recovery”) [25].

### 2.9. Data Analysis

Demographic characteristics and the distribution of the study outcome measures pre- and post-intervention were reported as the mean and standard deviation (SD) and/or the median, as well as the frequency distribution.

The Wilcoxon signed-rank test was applied to examine an improvement from pre- to post-intervention for the primary and secondary outcome measures in both groups. Group comparisons were assessed with the Mann–Whitney U-test. The Kolmogorov–Smirnov test and the Shapiro–Wilk test were used to check the data distribution.

A *p*-value of less than 0.05 was deemed statistically significant. The problem of multiplicity (to avoid the Type I error) that results from multiple comparisons, such as for the FMA and the SIS scales, was solved with the Bonferroni correction method. For this reason, when interpreting the *p*-values, *p* ≤ 0.01 instead of *p* ≤ 0.05 was considered statistically significant.

IBM SPSS-statistics^®^ (IBM Armonk, New York, NY, USA) for Windows version 24 and Microsoft Office Excel (https://www.microsoft.com (accessed on 3 November 2020)) were used to analyze the data. 

### 2.10. A-Posteriori Power Calculation

An a-posteriori power calculation was performed with G*Power 3.1 (Duesseldorf, Germany) [26], applying the Wilcoxon signed-rank test to matched pairs. Based on the data of the pre-intervention FMA-AS total score (mean = 33.5 ± 11.9) and the post-intervention FMA-AS total score (mean = 40.7 ± 10.9) in the IG, with a correlation between groups (*r*) = 0.984, *n* = 5, an alpha level of 0.05, and two-sided testing, a power of 99% was reached.

## 3. Results

### 3.1. Participant Characteristics

Participants were recruited between 8 August 2018 and 9 January 2020. The last participant completed the study on 29 January 2020.

Eleven of the 16 patients screened met all inclusion criteria and were included in the study. With respect to the proof-of-concept design, one patient who exceeded the defined inclusion criteria of falling into the early or late post-acute phase of 1–6 months by 539 days post stroke was included to evaluate the possibility of improvement even in the case of a chronic patient. Six participants were randomly assigned to the intervention group (IG) and the other six participants were assigned to the control group (CG). All 12 participants completed the study per protocol.

Participant demographics in the two groups were comparable except for the mean number of days since the stroke (“time since stroke”) before study entry; time since the stroke was observed to be longer in the CG due to the inclusion of one chronic patient; see Table 1. The time since the stroke without this chronic case was comparable (mean = 69 (±SD 14) for the CG).

The net intervention time was comparable (IG: mean = 23.5 ± 2 min; CG: mean = 24 ± 1 min). The pulse intensity (mA) was required to elicit a sufficient and possibly visible grip motion of the affected limb ranged 7–59 mA (mean = 16 ± 30 mA).

### 3.2. Therapy Effects: Primary Outcomes

In the IG, all participants improved pre- to post-intervention in the FMA-AS total score and its subgroups. This improvement can especially be observed for the FMA-AS total score with a mean difference of +7.17; for the FMA-AS A (mean difference of +4.00), and for the FMA-AS C (mean difference of +2.33). In the CG, the improvement pre- to post-intervention was most apparent for the FMA-AS total score (average difference of +6.33) and for the subgroup score FMA-AS C (average difference of +2.33); see Table 2.

In the group comparison, participants in the IG who all received the EMG-MES add-on to treatment, acquired greater apparent gain in the FMA-AS total score and the FMA-AS A subscore. The apparent gain is the difference between the group mean differences = diff(IG) − diff(CG) in Table 2. For the IG, the results were as follows: FMA-AS total score (apparent gain = +0.84) and the FMA-AS A subscore (apparent gain = +1.83).

### 3.3. Therapy Effects: Secondary Outcomes

Descriptive results (mean ± SD) of the BBT and the SIS recorded pre- and post-intervention in both groups are shown in Table 3.

For the BBT, both groups improved on the affected side (IG: mean = 7.17; CG: mean = 9.67), with the CG showing a larger improvement compared to the IG (mean group difference = diff(IG) − diff(CG) = −2.5).

For the SIS, participants in the IG improved from pre- to post-intervention in all the scales that were tested. The range of the mean improvement for all scales was 10.0–19.2 points. Similarly, in the CG, the range of the mean improvement for all scales was 1.04–15.4 points (Table 3).

For group comparison, the same calculation was carried out for Table 3. The participants in the IG group who all received the EMG-MES add-on to treatment showed a higher improvement in SIS Strength (mean group difference = diff(IG) − diff(CG) = +8.96), SIS ADL (mean group difference = +3.75), SIS Mobility (mean group difference = +4.64), SIS Hand function (mean group difference = +0.83), and SIS Stroke recovery (mean group difference = +13.33) compared to the CG. 

Two of the six participants in the IG reached the first target height (6 cm), two participants reached the second target height (12 cm), and two participants reached the third target height (18 cm), without any assistance from the therapist, by the end of the intervention. Not only hand and elbow but also shoulder activity improved.

### 3.4. Adverse Events

No adverse events or negative effects were reported in this study. Several participants in both study groups reported mild shoulder pain from the beginning of study procedures. The pain did not increase but even decreased during the intervention in some cases.

## 4. Discussion

Functional multichannel neuromuscular electrostimulation can be considered to induce grasp-release or finger-hand extension and shoulder-elbow function with the training of selective movements and activities of daily living. It can also enhance the recovery of selective movements in arm paresis after stroke [27]. Some authors describe a better outcome in intensive task-oriented single channel EMG-FES compared to classical single-channel electrical stimulation or voluntary movements in chronic stroke patients [10].

The data from a preliminary trial are reported here to investigate the feasibility and effects of two different types of ES on the recovery of CVM in selected subacute stroke patients. Our main interest was to test if the complex interventions and assessment procedures associated with cNMES and EMG-MES can be implemented into a daily routine for in-patient stroke rehabilitation.

In previous studies, daily ES treatment times of 45–60 min have been described as necessary to achieve certain beneficial effects, but negative side effects such as muscular fatigue were reported to occur after 10–15 repetitions on average for each movement pattern [11,12]. In this preliminary trial, it was also possible to demonstrate that EMG-triggered multichannel electrical stimulation with a net intervention time of 23.5 min was well tolerated by the study participants and well-integrated into the setting of an in-patient stroke rehabilitation clinic.

As expected, both groups improved in arm and hand CVM as measured with the FMA-AS. A noticeably higher gain for the IG group (the EMG-MES group) was found in the pre-/post-intervention comparison but due to the small number of participants and due to multiple testing, there were no significant changes. For the BBT, where individual task performance was measured, both groups improved significantly but there was no significant difference between the mean BBT scores for the IG and the CG in the group comparison.

Higher activity of daily living (ADL) in patients was found for the EMG-MES intervention, as quantified by the SIS. This is attributed to active patient involvement and task-oriented training, which seem to influence CVM recovery both in a subacute and chronic state of stroke recovery [7].

In this study, a sham control group was deliberately omitted because even subsensitive sham stimulation can elicit afferent sensory effects in the somatomotor cortex [28,29]. For this reason, a control group with established cNMES was chosen. The use of two established therapeutic ES methods (EMG-MES and cNMES) with a stimulation paradigm above the motor threshold was tested in this proof-of-concept study, focusing on the combination of multi-joint activities and the task-oriented problem-solving approach of the intervention group. This article should provide additional insight into this topic.

It should also be taken into account that cNMES, applied in the control group, is an intervention that is based on substantial amounts of data reported in the literature [5,30,31,32] and thus, also makes therapeutic effects likely, as can be observed in the FMA-AS subscale B: wrist (Table 2).

### 4.1. Limitation of the Study

In this preliminary trial on feasibility, there was no placebo or sham stimulation group in which participants receive a treatment option with a mesh glove or TENS stimulation technique. These techniques are known to have effects on sensory representation only, but no direct effect on the development of CVM or muscle endurance. Introducing a placebo or sham stimulation group would allow to the distinction of spontaneous recovery of CVM from the additional benefits of cNMES and EMG-MES, respectively. Since spontaneous recovery is to be expected in this phase of stroke rehabilitation, a control group should be planned for any subsequent trial; the control group would receive the mesh glove or TENS stimulation technique in addition to either cNMES or EMG-MES. 

The better performance of the intervention group may be due to the different treatment setup between the two groups by various confounding variables, such as the use of four pairs of electrodes, the arbitrary EMG triggering, or the active task-oriented therapy approach. As a more passive treatment approach, cNMES can consequently not be directly used for comparison. For this reason, both groups completed active task-oriented training in addition to the interventions. Although standard therapy was equal in both groups, we did not explicitly monitor whether all patients adhered to self-managed exercise program and resting periods in the same way. 

No specific assessment of pain/fatigue was part of the protocol. An assessment of pain/discomfort (such as the Spasticity-associated Arm pain scale, SAAPS; Ref. [33]) and an increase in muscle endurance functions should be integral parts of the subsequent study design.

In this feasibility study, there were no long-term follow-up assessments integrated to check the stability of the effects achieved with the interventions. Whether repetitive cNMES or task-oriented EMG-MES contributes to greater improvements in CVM and task performance remains unclear. 

The small number of cases does not allow a generally valid statement on the effectiveness of the interventions. These preliminary results should be confirmed by another study with a larger number of participants.

### 4.2. Directions for Future Work

Future studies might incorporate some of the study design components mentioned in the limitations of our study. Comparisons of shorter and longer intervention periods and durations of treatment sessions [12] and their effect on CVM, task performance, feasibility, and tolerability are of interest. Finally, follow-up assessments at 4- and 12-weeks post-intervention should be included in future study designs to assess the effects of different therapeutic methods.

In this preliminary RCT in carefully selected subacute stroke patients with moderate arm paresis, a three-week EMG-MES add-on to conventional treatment demonstrated a higher gain in CVM and self-reported daily activities, arm-hand use, and stroke recovery compared to a three-week add-on to conventional treatment of cNMES to the wrist only. EMG-MES appears to be an effective intervention that can be applied in routine clinical practice.

EMG-MES was well tolerated and did not lead to any side effects. EMG-MES might support the recovery of CVM in subacute stroke patients even better than cNMES.

The study design seems to be suitable for implementation in a larger trial to confirm these preliminary findings.

## Figures and Tables

**Table 1:** Demographic data and characteristics of the intervention and control groups.

		Mean (±SD)		
		IG	CG	Total
Sex	Male	4	4	8
	Female	2	2	4
Age		68 (7)	62 (9)	
Type of stroke	Ischemic	6	6	12
	Hemorrhagic	0	0	0
Lesion	Left	3	4	7
	Right	3	2	5
Affected hand	Dominant	3	4	7
	Not dominant	3	2	5
Degree of arm paresis	Severe to moderate	2	4	6
	Moderate to mild	4	2	6
Localization of infarction	Cortical	5	4	9
	Subcortical	1	2	3
Time since stroke	Days	63 (15)	147 (192) *	
Net intervention time	Min	23.5 (2)	24 (1)	

Abbreviations: IG—Intervention Group; CG—Control Group; ±SD—Standard Deviation. * Difference due to the inclusion of one chronic patient. Time since stroke without the chronic case was comparable (mean = 69 (±SD 14) for the CG).

**Table 2:** Descriptive results (mean ± standard deviation, SD) of the Fugl-Meyer Assessment Arm Section (FMA-AS) total score and scores on the subscales A, B, C, D.

IG (*n* = 6) CG (*n* = 6) Total (*n* = 12)	Mean (±SD)			Wilcoxon Signed-Rank Test ***		Mann–Whitney U-Test ****	
	Pre	Post	Diff	Z-Value	*p*-Values (2-Sided)	U-Value	*p*-Values (2-Sided)
FMA-AS total							
IG	33.5 (11.9)	40.67 (10.9)	7.17 (2.2)	−2.232	0.026 *	14.500	0.926
CG	31.67 (5.4)	37.67 (5.2)	6.33 (4.5)	−2.003	0.045 *		
FMA-AS A							
IG	18.83 (5.9)	22.67 (5.7)	4.00 (2.2)	−2.226	0.026 **	12.000	0.576
CG	19.50 (2.3)	21.67 (4.2)	2.17 (3.4)	−1.476	0.140		
FMA-AS B							
IG	5.00 (1.9)	6.17 (1.3)	1.17 (1.6)	−1.604	0.109	7.000	0.129
CG	5.00 (2.1)	6.50 (2.7)	1.67 (1.2)	−2.041	0.041		
FMA-AS C							
IG	7.33 (3.5)	9.17 (3.7)	2.33 (2.1)	−2.041	0.041 **	14.500	0.926
CG	6.67 (2.7)	8.83 (2.8)	2.33 (2.1)	−1.897	0.058		
FMA-AS D							
IG	2.33 (2.1)	2.67 (2.5)	0.33 (0.8)	−1.000	0.317	15.000	1.000
CG	0.50 (1.2)	0.50 (1.2)	0.00 (0.0)	0.000	1.000		

* significant improvement pre to post *p* = 0.05. ** higher improvement pre to post. Due to multiple comparisons the significance level was increased according to Bonferroni from *p* = 0.05 to *p* = 0.01. *** difference between pre and post. **** difference between IG and CG.

**Table 3:** Descriptive results (mean ± standard deviation, SD) of the Box and Block Test (BBT) and Stroke Impact Scale (SIS).

IG (*n* = 6) CG (*n* = 6) Total (*n* = 12)	Mean (±SD)			Wilcoxon Signed-Rank Test ***		Mann-Whitney U-Test ****	
	Pre	Post	Diff	Z-Value	*p*-Values (2-Sided)	U-Value	*p*-Values (2-Sided)
BBT affected hand							
IG	26.00 (11.4)	32.33 (13.1)	7.17 (4.1)	−2.023	0.043 *	14.000	0.520
CG	16.83 (7.1)	27.00 (3.6)	9.67 (5.6)	−2.207	0.027 *		
SIS Strength							
IG	55.00 (22.3)	64.58 (6.5)	10.00 (25.2)	−0.730	0.465	12.000	0.579
CG	48.96 (16.5)	51.04 (19.1)	1.04 (12.8)	−0.557	0.577		
SIS ADL/IADL							
IG	51.67 (17.3)	70.83 (18.1)	19.17 (14.4)	−2.207	0.027 **	16.000	0.744
CG	50.42 (11.5)	65.83 (12.5)	15.42 (5.3)	−2.226	0.026		
SIS Mobility							
IG	64.33 (38.2)	80.08 (26.02)	15.75 (18.9)	−2.023	0.043 **	12.000	0.584
CG	75.46 (18.4)	86.57 (10.8)	11.11 (11.5)	−1.892	0.058		
SIS Hand function							
IG	30.83 (27.1)	45.83 (23.5)	15.00 (14.8)	−2.023	0.042 **	11.000	0.459
CG	22.50 (21.6)	36.67 (40.5)	14.17 (19.9)	−1.473	0.141		
SIS Recovery							
IG	49.17 (11.1)	66.67 (13.7)	17.50 (10.8)	−2.214	0.027 **	7.500	0.086
CG	47.50 (18.1)	51.67 (20.2)	4.17 (9.1)	−1.063	0.288		

* significant improvement pre to post *p* = 0.05. ** higher improvement pre to post. Due to multiple comparisons, the significance level was increased according to Bonferroni from *p* = 0.05 to *p* = 0.01. *** difference between pre and post. **** difference between IG and CG.

## Data Availability

Data available from the corresponding author upon reasonable request due to privacy restrictions.
